# NEATTILL: A simplified procedure for nucleic acid extraction from arrayed tissue for TILLING and other high-throughput reverse genetic applications

**DOI:** 10.1186/1746-4811-6-3

**Published:** 2010-01-26

**Authors:** Yellamaraju Sreelakshmi, Soni Gupta, Reddaiah Bodanapu, Vineeta Singh Chauhan, Mickey Hanjabam, Sherinmol Thomas, Vijee Mohan, Sulabha Sharma, Rajeswari Srinivasan, Rameshwar Sharma

**Affiliations:** 1 School of Life Sciences, University of Hyderabad, Hyderabad 500 046, India

## Abstract

**Background:**

TILLING (Targeting Induced Local Lesions in Genomes) is a reverse genetics procedure for identifying point mutations in selected gene(s) amplified from a mutagenized population using high-throughput detection platforms such as slab gel electrophoresis, capillary electrophoresis or dHPLC. One essential pre-requisite for TILLING is genomic DNA isolation from a large population for PCR amplification of selected target genes. It also requires multiplexing of genomic DNA isolated from different individuals (pooling) in typically 8-fold pools, for mutation scanning, and to minimize the number of PCR amplifications, which is a strenuous and long-drawn-out work. We describe here a simplified procedure of multiplexing, NEATTILL (Nucleic acid Extraction from Arrayed Tissue for TILLING), which is rapid and equally efficient in assisting mutation detection.

**Results:**

The NEATTILL procedure was evaluated for the tomato TILLING platform and was found to be simpler and more efficient than previously available methods. The procedure consisted of pooling tissue samples, instead of nucleic acid, from individual plants in 96-well plates, followed by DNA isolation from the arrayed samples by a novel protocol. The three variants of the NEATTILL procedure (*vast, in-depth *and *intermediate*) can be applied across various genomes depending upon the population size of the TILLING platform. The 2-D pooling ensures the precise confirmation of the coordinates of the positive mutant line while scanning complementary plates. Choice of tissue for arraying and nucleic acid isolation is discussed in detail with reference to tomato.

**Conclusion:**

NEATTILL is a convenient procedure that can be applied to all organisms, the genomes of which have been mutagenized and are being scanned for multiple alleles of various genes by TILLING for understanding gene-to-phenotype relationships. It is a time-saving, less labour intensive and reasonably cost-effective method. Tissue arraying can cut costs by up to 90% and minimizes the risk of exposing the DNA to nucleases. Before arraying, different tissues should be evaluated for DNA quality, as the case study in tomato showed that cotyledons rather than leaves are better suited for DNA isolation. The protocol described here for nucleic acid isolation can be generally adapted for large-scale projects such as insertional mutagenesis, transgenic confirmation, mapping and fingerprinting which require isolation of DNA from large populations.

## Background

Several strategies for crop improvement for increased yield, better agronomic or novel traits, improved resistance to diseases and pests, to meet demand for biofuels and secondary metabolites for pharmaceutical or industrial purposes have been targeted by breeders and plant biologists. These strategies range from conventional selection processes, mutagenesis and breeding, to biotechnological approaches such as genetic transformation, RNA interference, insertional mutagenesis or targeted manipulation by zinc finger nucleases and rapid trait development system (RTDS) [1-3].

In the post-genomics era, the major impetus is on annotation of the genome sequence resources generated and assigning functions to individual genes. In general, the availability of such genome sequence resources in several plant species has given researchers an opportunity to shift from the traditional forward genetics approach to the reverse genetics strategies, which unlike the former approach essentially correlate the genomic sequence data to the phenotypic traits. The daunting task of deciphering the functions of newly discovered genes in various plants has ushered the researchers into the area of functional genomics and the focus has shifted to tools which can perform in high throughput manner.

In this direction one such tool developed is Targeting Induced Local Lesions IN Genomes (TILLING) [4-6], which essentially integrates the conventional mutagenesis approach with high throughput mutation detection. It involves generating a large mutant population by treatment of seed or pollen (in case of plants) with alkylating agents such as EMS (ethylmethane sulphonate) or ENU (ethylnitrosourea) with the aim of saturating the whole genome with mutations. This class of mutagens causes large number of random point mutations in the genome, thus theoretically multiple alleles of any gene can be obtained in the population. For TILLING, genomic DNA is isolated from M_2 _plants (5,000-10,000), pooled (2-8 folds) and is amplified by PCR with a set of differentially labeled fluorescent primers. The PCR products are denatured and renatured and are digested with a S1/P1 endonuclease, CEL I, which cuts the DNA heteroduplexes specifically at the site of mismatch. The digested products after electrophoresis are visualized using LI-COR Analyzer, denaturing dHPLC or even agarose gels [5-9].

One essential feature of TILLING is that it needs pooling of genomic DNA of mutant with wild type thus allowing generation of mismatches in heteroduplexes. Therefore, procedure of TILLING utilizes the multiplex approach i.e. pooling at least two DNA samples, prior to PCR amplification. Since mutagenesis is a random event, it obviates the need of using wild type DNA for pooling and different numbers of individuals upward of two can be pooled for mutation detection. The upper limit of pooling is determined by the efficiency of mutation detection procedure. One obvious advantage of pooling is the considerable reduction in the number of PCR amplifications to be carried out for mutation detection. Moreover, pooling also reduces the cost of consumables and the time needed for mutation scanning. In most species (Arabidopsis, maize, lotus, barley, wheat, Drosophila, soybean, sorghum, Phytophthora) where TILLING was used to detect point mutations, the samples were pooled after DNA extraction, prior to PCR amplification step of TILLING [7,10-17]. However, pre-amplification pooling of genomic DNA is not a strict requisite, for example in zebrafish instead of genomic DNA, four individual PCR-amplified samples were pooled prior to CEL I digestion [18].

In general, the choice of pooling is determined by striking a balance between total number of PCR reactions to be carried on population and chances of detection of mutations in the pool. Different groups have followed various levels of pooling i.e. four, six, eight or even higher folds of DNA pooling, however, the higher folds of pooling beyond 8-fold pool, reduce the chances of mutation detection. Almost all the methods previously described for TILLING rely on isolation of individual DNA samples (1,000-15,000) followed by pooling (one/two dimensional) [19,20]. The harvesting and arraying of large number of samples and subsequent DNA isolation is a protracted process and often takes days or even weeks to generate primary DNA resource for a given population. This effort and time is compounded by the obligatory necessity of DNA dilution, equalization, and x-fold pooling to reduce the number of samples for PCR amplification. Nonetheless, these steps are essential for TILLING and require considerable inputs in terms of time and labor.

Here we describe a simplified 2-dimensional pooling strategy, NEATTILL- Nucleic acid Extraction from Arrayed Tissue for TILLING, and a modified high throughput DNA isolation protocol for tomato. Our protocol circumvents isolation of DNA from individual plants by pooling of tissue from different plants followed by DNA isolation from pooled arrayed samples. The scanning of mutations in 2-D pooled plates allows simplified identification of mutant lines as a mutation detected in the row-pooled plate is also represented in the complementary column-pooled plate, allowing direct identification of mutant lines. The pooling of tissue prior to DNA isolation reduces the total number of extractions with added benefits of cost reduction and time saving.

## Results and Discussion

### Comparative evaluation of DNA isolation protocols

**Figure 1:**
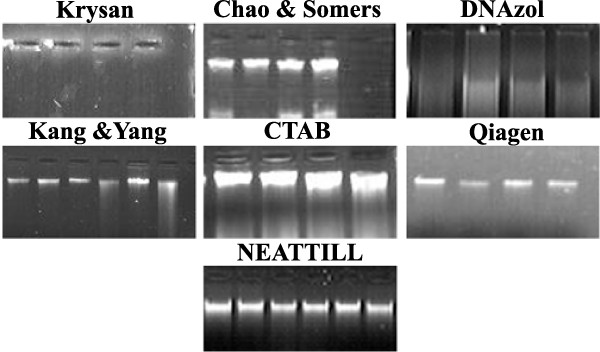
**Qualitative comparison of DNA isolated using different protocols on agarose gel electrophoresis**. DNA was isolated from tomato leaf tissue by methods descibed by Krysan *et al *[23], Kang and Yang [24], Chao and Somers [25], CTAB [21], DNAzol [22], Qiagen kit [26] and NEATTILL respectively.

With the aim to develop a cost effective, efficient and high throughput method for genomic DNA isolation from plants we compared several published protocols in terms of their output and efficiency using tomato leaves. Reagents such as CTAB [21], DNAzol (Life Technologies) [22] or protocols developed by Krysan *et al *[23], Kang and Yang [24], Chao and Somers [25] were used for DNA isolation using Eppendorf tubes as well as 96 deepwell plate formats. These protocols were compared on the basis of five criteria as given in Table 1 with a commercial kit supplied by Qiagen (DNeasy Plant Mini Kit) [26]. Suitability of the protocol for DNA isolation was judged by its scalability (i.e. whether isolation could be done singly in tubes or in 96 deepwell plates), yield and quality of isolated DNA and investment in terms of resource (starting material), time and money. Figure 1 and Table 1 show that genomic DNA of good quality (260/280 ratio of 1.9) and quantity (up to 15 μg/100 mg tissue) could be isolated using the protocol of Chao and Somers [25]. While CTAB procedure also gave a good quality DNA (260/280 ratio of 1.89), this procedure is not adaptable for 96 deepwell plate format as it involves organic extractions with chloroform: isoamyl alcohol. First it is difficult to do phase separation in 96 well plates and second, organic solvents loosen the silicon rubber sealing mats of the 96 deepwell plates during inversions leading to sample loss and cross-contamination. The protocol of Krysan *et al*. [23] although most rapid and least expensive, was found not suitable for DNA isolation from tomato leaves. The usage of both commercial reagent, DNAzol and Qiagen kit yielded relatively less amount of DNA (3.5 μg/100 mg and 5 μg/100 mg) with 260/280 ratio of less than 1.8 (~1.16/1.63). The low 260/280 ratio of Qiagen DNA preparations can be explained as the procedure is based on silica matrix and uses a strong chaotropic salt such as guanidine hydrochloride for purification step. Incomplete removal of salts from the DNA preparations tend to lower the 260/280 ratio below 1.8, although these interfering salts usually do not interfere in PCR amplification reactions. Among the protocols evaluated the Chao and Somers [25] protocol was selected for further improvement.

**Table 1:** Comparison of different DNA isolation protocols

Protocol	Feasibility of isolation in 96 well plates	Yield (μg/100 mg tissue)	A_260/280 _ratio	Time required (h)	Cost (in USD/100 samples)*
**Krysan *et al ***[23]	Yes	Nil	Nil	1	6

**Kang and Yang **[24]	No	10	1.12-1.37	1-3	10-20

**Chao & Somers **[25]	Yes	15	1.9	3	9

**CTAB **[21]	No	6.5	1.89	4-5	12

**DNAzol **[22]	No	3.5	1.16	3	164

**Qiagen **[26]	Yes	5	1.63	2	364

**NEATTILL**	Yes	40	1.83	7	14

*The indicated cost is for 100 DNA preparations using Eppendorf tubes. DNA isolation with 96 deepwell plate using NEATTILL costs 40 USD/plate compared to 220 USD/plate with Qiagen protocol.

### Elimination of phenolics

**Figure 2:**
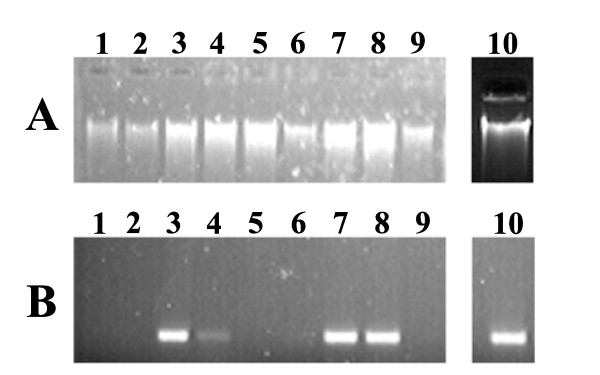
**Genomic DNA quality and PCR amplification of DNA isolated using different modifications A- Gel electrophoresis of DNA Lane**. 1: Tissue was homogenized in extraction buffer (0.1 M Tris-HCl, pH 7.5; 0.05 M EDTA, pH 8.0; 1.25% (w/v) SDS), Lane 2: Same as lane 1 with additional step of PCI (25:24:1). The DNA pellet after dissolving in 200 μl of milliQ water was extracted with an equal volume of PCI. The aqueous phase was collected and DNA was reprecipitated and dissolved in milliQ water, Lane 3: Same as lane 1 with inclusion of 2% (w/v) PVP and 0.2 M β-ME during extraction and PCI extraction was done as for lane 2, Lane 4: Same as in lane 3 but without PCI step, Lane 5: Same as in lane 3 but without β-ME, Lane 6: Same as lane 1 with inclusion of 2% (w/v) PVP during extraction, Lane 7: Same as lane 1 with inclusion of 30 mg PVPP and 0.2 M β-ME during extraction and PCI extraction as done for lane 2, Lane 8: Same as lane 1 with inclusion of 30 mg PVPP and 0.2 M β-ME during extraction, Lane 9: Same as lane 1 with inclusion of 30 mg PVPP during extraction, Lane 10: DNA isolated by Qiagen kit. **Abbreviations: **PCI-phenol:chloroform:isoamyl alcohol, β-ME-β-mercaptoehanol, PVP-Polyvinylpyrrolidone, PVPP-Polyvinylpolypyrrolidone. B- PCR amplification of isolated DNA. The quality of DNA was checked by PCR amplification of actin gene using forward primer 5'TAACCCAAAAGCCAATCGAG3' and reverse primer 5'AGCTTCCATTCCGATCATTG3'.

Though the yield of genomic DNA from tomato leaves was good using Chao and Somers protocol [25], the PCR amplification deteriorated on storage. The close examination revealed that DNA contained carryover of polyphenols which imparted a brown colour to the preparations and hindered with PCR amplification of target templates. It is known that phenolics are major secondary metabolites in plants which get oxidized during homogenization and impart dark colour to DNA preparations [27,28]. We therefore evaluated several possible steps that could remove the phenolics during tissue homogenization using above protocol. Several modifications (inclusion of PVP, PVPP, β-mercaptoethanol and extraction with phenol: chloroform: isoamyl alcohol) were done in various combinations. The untreated and treated DNA preparations were then checked for their quality by amplifying actin gene fragment as control (Fig. 2A, B). The preparations which included β-mercaptoethanol (which acts as a strong reductant by breaking intramolecular disulphide bonds in proteins and prevents oxidation of polyphenols) and PVPP (which helps in removal of polyphenols) during homogenization gave the high PCR amplification. Also, PCR amplification reaction obtained using a combination of β- mercaptoethanol and PVPP were comparable to the amplification obtained from DNA isolated using Qiagen kit (Fig. 2B). Additional modification was the treatment with RNase to remove contaminating RNA in DNA preparations.

**Figure 3:**
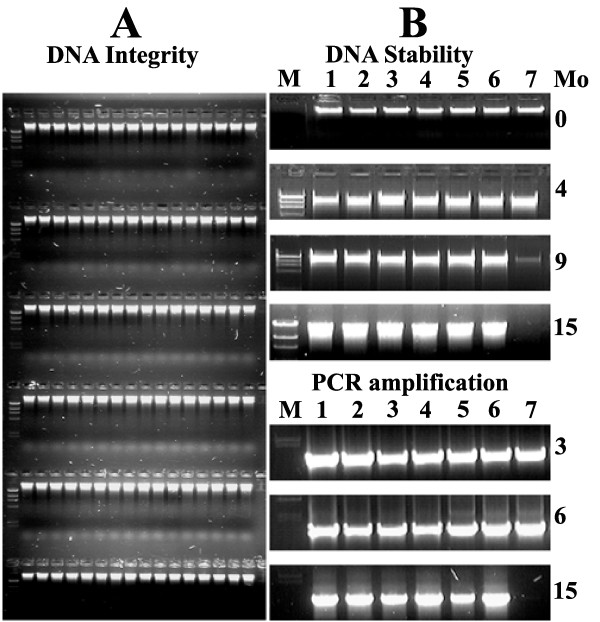
**Evaluation of DNA quality and stability during long-term storage at 4°C**. A- Integrity of DNA isolated in 96 deepwell plate after EtBr staining on agarose gel; B- Long term stability (Top panel) and PCR amplification (bottom panel) of nucleic acids. The DNA afer isolation (labeled **1-7**) was suspended in either TE/RNase (Lane 1-6) or water (Lane 7) and stored at 4°C. At different time periods (0, 4, 9 and 15 months) from isolation DNA was checked for integrity using agarose gel electrophoresis. The quality of DNA was checked by PCR amplification of actin gene (primers as in Fig. 2) using aliquots from stored DNA at 3, 6 and 15 months. M-Markers, Mo-Months.

After incorporation of the above modifications, we used the protocol in 96 well plate format for DNA isolation using juvenile leaves and cotyledons (data shown only for cotyledons). We obtained a nearly uniform isolation of DNA with an average yield of 20-30 μg DNA per well (Figure 3A). We also found that amount of tissue for DNA isolation should be restricted to 100 mg per well. Any further increase of tissue weight resulted in poor quality of DNA. However the above method can also be adopted for DNA isolation in tubes. We found that usage of Eppendorf tubes gave higher yields of DNA (up to 40 μg/100 mg) as tubes could be centrifuged at much higher speeds than the plates.

The long-term stability of isolated DNA is paramount for TIILING as genomic DNA is used for several years. We found that DNA suspended in TE (10 mM Tris pH 7.5 and 1 mM EDTA pH 8.0) supplemented with RNase (32 μg/ml) (Till *et al *[19]) was stable and retained its quality and PCR amplification even after storage at 4°C for 15 months. In contrast, the DNA suspended in water degraded over time and did not show any PCR amplification after 15 months (Fig. 3B). For long-term storage, we store DNA suspended in TE with RNase in -20°C freezers. With these modifications for DNA isolation and storage we used the NEATTILL protocol for high throughput DNA isolation from tomato cotyledons.

### Choice of tissue for arraying and DNA isolation

Higher eukaryotes offer several possibilities for the starting tissue material for DNA isolation. Choosing the right tissue for arraying and DNA isolation is critical in the overall NEATTILL procedure as it generates the genomic DNA resource for TILLING. Factors such as easy availability, feasibility of culture (in case of micro-organisms and cell lines) and growth (in case of plants), minimum post-collection manipulation with samples (such as specific treatments or tissue cleaning, cutting into pieces), age of the tissue sample, absence of secondary metabolites (especially relevant in case of plants) and DNA stability during long term storage can influence one's choice of tissue. Most often genomic DNA is isolated from leaves of plants grown in field or green house. We found that for tomato DNA isolated from mature leaves was of inferior quality due to the presence of secondary metabolites (especially, anthocyanins and polyphenolics). The usage of emerging juvenile leaves, which had less secondary metabolites, yielded better quality DNA. Relatively superior quality DNA was obtained from young cotyledons with minimal interference from secondary metabolites. One advantage of using cotyledons as starting material is that DNA isolation can be done using culture room grown plants at any time of the year.

### NEATTILL: Simplified pooling strategy of arraying of tissue samples

In addition to modified DNA isolation protocol, we adopted pooling of tissue prior to extraction of DNA which is advantageous, especially, while handling large number of plants. First, it causes an 8 fold reduction in number of DNA preparations to be handled; second, by reducing the number of steps, it also reduces the chances of contamination by nucleases. To ensure that the DNA from each individual plant is equally represented, the tissue of almost equal weight or size was selected. The total amount of tissue to be harvested for DNA extraction is determined by the overall volume available for tissue homogenization and processing in the deepwell plate and sample weight/extraction buffer ratio. In case of tomato we used equal sized cotyledons and the total weight of 8 cotyledons taken per well was about 80-100 mg, which could be homogenized in 2 ml volume available in each well of deepwell plates. For pooling of cotyledons, we germinated 8 seedlings per mutant line in 96 well plates and one seedling per line was pooled. On completion of pooling of all the mutant lines in the population, we again harvested the seedlings as in the previous case and subjected it to second round of pooling to generate backup plates.

**Figure 4:**
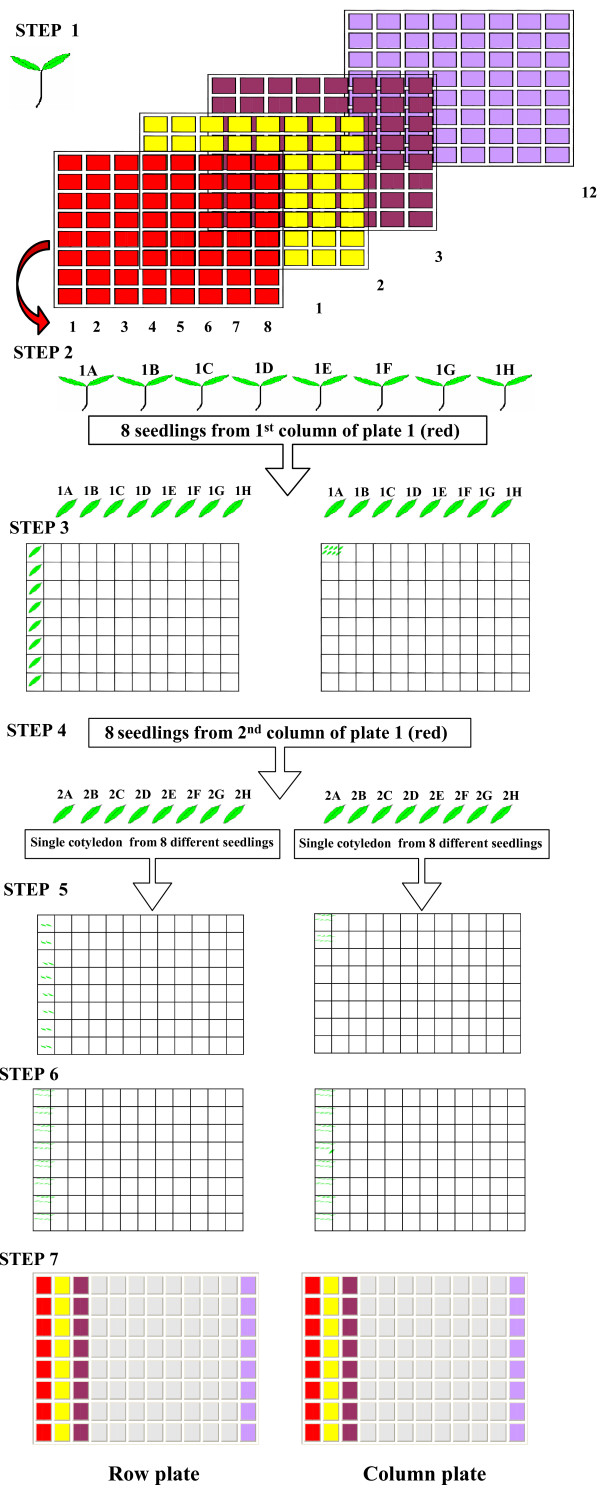
**Two-dimensional pooling of the cotyledons in 96-well plates**. Step 1: Twelve 8 × 8 grids were prepared (as depicted by numericals 1, 2, 3..... 12 and colored red, yellow, brown.... lavender, respectively,). Step 2: The eight seedlings arrayed in column 1 of the first grid were removed and arranged linearly. The right and the left cotyledons of the seedlings were excised and arranged in the same order as present in the column of the grid from which they were removed. Step 3: In two new deepwell plates, the cotyledons were placed in the wells as shown in the Figure. One set of cotyledons was placed in 1A-1H wells in the ROW plate whereas the other set of cotyledons was placed in 1A well of COLUMN plate. Step 4: Eight seedlings from 2A-2H of plate 1 (red colored) were removed and their cotyledons excised as in step 2. Step 5: One set of cotyledons was placed in 1A-1H of ROW plate whereas the other set was put in 1B well of COLUMN plate. Step 6: Similarly the seedlings were removed from columns 3, 4, 5, 6, 7 and 8 and pooled as described steps 2 and 3 in different wells. After pooling the seedlings of eight columns, both the ROW and COLUMN plate had eight cotyledons each in 1A-1H wells. Step 7: The whole procedure was repeated for the second, third....twelveth grid (as shown with yellow, brown ....lavender colors in Figure) to generate eight pooled ROW and COLUMN plates.

**Figure 5:**
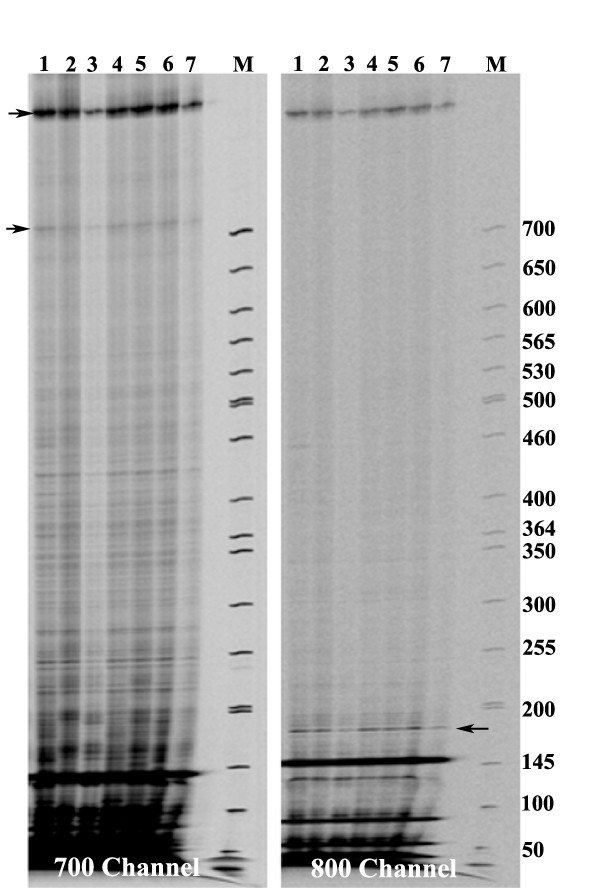
**Identification of cleaved fragments of *hp1 *mutant after CEL I digestion in Licor 4300 DNA Analyzer**. Positive control DNA bearing *hp1 *mutation was laced with eight-fold pooled DNA which was isolated by NEATTILL procedure as described in the text. The expected position of the CEL I cleaved fragments are 718 bp (bottom left arrow) in 700 channel and 183 bp (right arrow) in 800 channel which were detected in all replicates. These cut fragments add up to 901 bp (top left arrow) (total amplicon length of *hp1 *gene). M on right hand side of the two images shows 50-700 bp size standards. The fragments detected specially in the lower portion of either channel images represent the background fragments usually observed in TILLING runs. However these can be distinguished from the CEL I cleaved fragments as these fragments do not show the complementary fragment in the either channel, a characteristic of CEL I cleaved fragments.

The cotyledon arraying strategy (Fig. 4) simultaneously generated a row and a column plate each carrying 768 mutant lines. The placement of the two cotyledons of a single seedling in the row and column plates led to direct identification of the precise mutant line during mutation screening from 8-fold pools. In other words, for every mutant pool identified in a plate (e.g. row) during screening, the exact mutant line was subsequently identified while screening its complementary plate (column). Once a mutation was identified, seeds from the M_2 _seed packet of that mutant line were sown. The individuals were then examined for the presence of mutation and were sequenced. The efficiency of above method in terms of mutation detection was ascertained using tomato *hp1 *mutant as a control [29] (Fig. 5).

### Tissue arraying options

**Figure 6:**
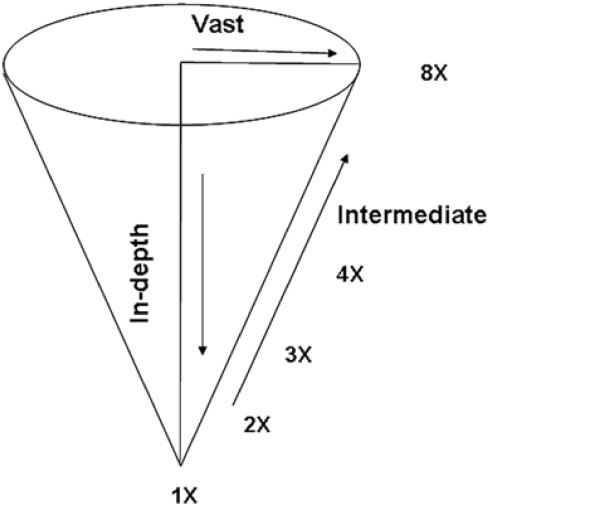
**Different pooling options for screening a population**. Vast - 8× pooling suitable for >5000 individuals in a population; In-depth- 1× pooling (6-8 individuals of one mutant line) suitable for < 1000 individuals in the population; and intermediate- 2×/3×/4× pooling to strike a balance between vast and in depth screening.

The tissue arraying gives three options of representing mutant lines of the whole population for screening depending upon the size of the mutagenized population. Large populations having >5000 up to 15000 individual mutant lines can be pooled by combining tissue samples from 6-8 mutant lines to generate 6×-8× pooled plates as described above in tomato. Population sizes smaller than 1000 individuals can be subjected to ***in-depth ***scanning in which 6-8 individuals of the same mutant line can be pooled together [30]. This improves the chances of detecting the mutation while scanning the whole population particularly for organisms which have "genetically effective cell number" more than one. However, the number of plates to be screened also increases, so this option is practically feasible only with small population (Fig. 3). Another manipulation, termed as ***intermediate ***scanning, at the level of fold pooling (2×-8×) can also be attempted (Fig. 6). Here pooling can be done by combining 2 lines with 4 individuals each (2×) or 3 lines with 2 individuals each (3×) or 4 lines with 2 individuals each (4×) as detailed in Figure 3. It is left to the discretion of the researcher to choose either of the options [to attempt ***vast ***scanning (8×) or ***in-depth ***scanning (1×)] or strike a balance between the two options by manipulating fold pools (2×-4×) (Fig. 6).

### A cost effective protocol for high throughput DNA isolation

**Figure 7:**
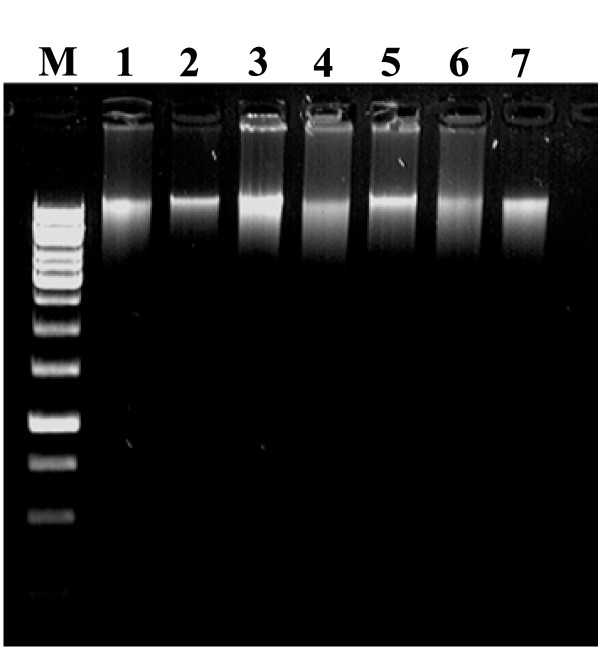
**DNA isolated from different plant species**. Agarose gel electrophoresis of DNA extracted using NEATTILL from different plant species. Lanes; 1: Tomato; 2: *Arabidopsis*; 3: *Jatropha*; 4: *Citrus*; 5: Sunflower; 6: *Ricinus*; 7: *Indigofera*. M- marker

The described NEATTILL protocol accomplishes germination of seedlings, arraying of cotyledons, extraction of DNA, quantification and equalization in approximately two weeks time (germination: 7-12 days; arraying of cotyledons: 1 day; extraction of DNA: 1 day; quantification and equalization: 1 day). The arraying, DNA extraction, quantification and equalization merely take 3-4 days time. DNA from about 3072 samples (four 96 well plates) can be conveniently isolated by this protocol in 6-7 h time. Since all reagents and consumables can be directly purchased, compared to the commercial kit and reagent (Qiagen, DNAzol) it is more economical (Table 1). The described protocol is not restricted to tomato and it yielded high quality DNA using juvenile leaves from different plant species namely *Arabidopsis*, *Jatropha*, *Citrus*, sunflower, *Ricinus *and *Indigofera *(Fig. 7).

### Limitations

NEATTILL, although offers the flexibility of scanning populations in 3 ways: vast, in-depth or intermediate, has one limitation that the fold pooling of DNA is fixed. However, when DNA is isolated separately from individuals, one can try various levels of pooling (2×, 3×, 4×, 5×, 6× or 8×) at any point of time but this flexibility is not possible with tissue pooling. Therefore, fold pooling which works best in a particular organism has to be determined before attempting NEATTILL using a known mutation as a positive control.

## Conclusions

Large scale TILLING projects require huge investments in terms of infrastructure, reagents, consumables, time and labor for generation and subsequent analysis of mutagenized populations. To make the procedure high throughput, multiplexing of samples is preferred. We developed a procedure for multiplexing in which tissue samples are combined instead of nucleic acid as previously used for TILLING. The procedure, NEATTILL, saves time, lowers the reagent costs by up to 90% and is less labour intensive as the number of DNA samples is reduced. Consequently, less number of samples has to be equalized and pooled. The three variants of NEATTILL- *vast, in-depth and intermediate *can be attempted according to the population size or fold pooling giving the flexibility to represent sufficient number of individuals per mutant line for screening.

The choice of tissue for DNA extraction is critical for obtaining best quality DNA. In case of tomato, cotyledons yielded better quality DNA than leaves. Cotyledon or juvenile tissue pooling from seedlings can be applied to other plant species too. After tissue collection, the seedlings could be transplanted in field for phenotypic characterization and this has an added advantage of having DNA from the mutant line which is also phenotypically characterized. The 2-D pooling generates complementary plates (row and column) simultaneously. The screening of these complementary plates gives the exact co-ordinates of the positive mutant line and setting of separate reactions to deconvulate a mutant pool is avoided.

## Methods

### Plant material and Consumables

EMS mutagenized M_2 _seeds of tomato (*Solanum Lycopersicon*) *cv*. Arka Vikas (Arka vikas seeds were originally obtained from Indian Institute of Horticulture Research, Bangalore) or *cv*. M82 raised by Menda *et al *[31] were used for the study. All plasticware including deepwell plates (2 ml) were procured from Axygen Limited, India. The equipments used were capable of accommodating 96 well plasticwares: high speed centrifuge (Evolution RC, Sorvall with SH-3000 swinging bucket rotor capable of accommodating 4 deepwell plates at a time), 96 well pipettor (PP550 DS, Apricot Designs), Mini Bead Beater (BioSpec Products Inc.); PCR machine (DNA Engine Tetrad 2, MJ Research), 4300 DNA Analyzer (Li-COR Biosciences). Other equipments commonly used in molecular biology laboratory were: horizontal gel electrophoresis system, gel documentation system (Alpha Imager™ 2200, Alpha Innotech), Nanodrop ND-1000 spectrophotometer, dry bath, water baths etc. All chemicals for DNA isolation were from Sigma-Aldrich and the organic solvents used in the procedure were from Qualigens Limited, India. Stock solutions for DNA isolation and TILLING were prepared in sterile MQ water (Milipore water purification system).

### Two dimensional 8-fold pooling of cotyledons

The pooling of cotyledons basically consisted of three steps:

#### Step A: Germination of seeds

Prior to sowing, the M_2 _seeds were surface sterilized in 2% (v/v) sodium hypochlorite for 15-20 min followed by washing under running tap water. Seedlings were grown in wells of 96 deepwell plates (2 ml) filled with Soilrite mixture. Prior to seed sowing, well bottoms were perforated to facilitate water uptake in Soilrite through bottom. The plates were kept in large plastic trays filled with 1 cm of water. In each 96-well plate, 8 seeds each of 24 individual mutant lines were sown by placing 2 seeds in sample wells using a pre-planned grid. In essence, for any single column of plate, in the first four well (A-D) seeds of *X *mutant lines and in next four wells (E-H) seeds of *Y *mutant line were sown. The seedlings were grown under white fluorescent light (100 μmol/m^2^/sec) in growth room at 25 ± 2°C till harvest. The usage of 8 seeds ensured the availability of sufficient number of cotyledons for tissue pooling at the time of harvest.

#### Step B: Harvesting of seedlings

Seedlings were harvested after full expansion of cotyledons usually 7-10 days from germination. Seedlings along with their roots were pulled from the wells and washed with water to remove adhering Soilrite to the roots. They were serially placed in another 96 deepwell plate (2 ml) filled with water in a preplanned 8 × 8 grid fashion. Twelve such 8 × 8 grids (Fig. 4, Step 1) were made to generate a '**row**' and a '**column**' 8× pooled plate. Pre-planned pooled grids were prepared prior to harvesting the seedlings and cotyledon pooling with numbers of each mutant lines entered manually in the grid. It is essential to ensure proper placement of seedlings and cotyledons during the process, and it is better conducted with two individuals.

#### Step C: Eight fold pooling of cotyledons in 2-D fashion

For pooling of cotyledons for DNA isolation, two sterile plates were set up, one for column pooling and second for row pooling. Since harvesting of the cotyledon was done in column wise fashion, a simplified procedure was adopted to ensure the correct pooling of cotyledons. The right cotyledon from all eight seedlings (arranged in a column in 8 × 8 grid plate, red in colour in Fig. 4, step 2) was excised and harvested to make a single column pool, and placed in well 1A of 'column' plate whereas left cotyledon from each seedling was placed in wells of column 1 A to H of the 'row' plate. The next harvest of right cotyledons generated 8-pooled well 1B of 'column' plate and left cotyledons were again placed in wells of column 1 A to H of the '**row**' plate, increasing number of cotyledons to two. The entire process of pooling batches of eight seedlings from 8 × 8 grids was repeated for 3^rd ^- 8^th ^columns of the 8 × 8 grid. At the end of total harvest of 8 × 8 grids, 8 cotyledons from grid were pooled in column fashion and 8 cotyledons were pooled in row fashion. Using 12 plates of seedlings we obtained two plates of cotyledons arrayed in row and column fashion (Fig. 4, Step 7). The pooled plates bearing cotyledons were sealed with rubber sealing mats and stored at -80°C freezer until DNA isolation.

#### Isolation of nucleic acids

Extraction of DNA was essentially carried out as described below. All pipetting was carried out using 96 well pipettor from Apricot Designs.

#### Step A: Grinding and disruption of tissue

Mechanical disruption of tissue (80-100 mg/8 cotyledons) with three steel balls of ~2 mm in diameter was done in Mini-Bead Beater for 2 min in the presence of 750 μl preheated (65°C) extraction buffer (0.1 M Tris-HCl, pH 7.5; 0.05 M EDTA, pH 8.0; 1.25% (w/v) SDS) containing 0.2 M β-mercaptoethanol and 20 mg of insoluble polyvinylpolypyrrolidone (PVPP). The plate was then incubated at 65°C for 30 min for cell lysis.

#### Step B: Removal of contaminating RNA

The plate was brought to room temperature by placing it on the lab bench for 5 min. The samples were incubated with RNase (53 μg/ml, Sigma) at this stage by adding 4 μl from 10 mg/ml stock and incubated at 37°C in a water bath for 30 min for removing the RNA from the preparations.

#### Step C: Separation of proteins and other cellular debris

Precipitation of the proteins was done by the addition of 400 μl of cold 6 M ammonium acetate. The plate was sealed with the sealing mat and the samples were mixed with ammonium acetate by repeated inversion of plates. Thereafter, plates were incubated at 4°C for 15 min followed by 30 min centrifugation at 4700 rpm in a swing out plate rotor (SH-3000) in a Sorvall RC Evolution centrifuge. The precipitated proteins along with other cellular debris were pelleted at the bottom and an aliquot from the clear supernatant (650 μl) containing DNA in aqueous phase was transferred to a new plate using 96 well pipettor.

#### Step D: Precipitation of nucleic acids and removal of residual salts

Equal volume (650 μl) of cold isopropanol was added to each well to precipitate DNA and plates were incubated at -20°C for at least 2 hrs. The plates were then centrifuged at 4700 rpm at 4°C for 30 min followed by two 70% (v/v) ethanol washes to remove traces of salts from the samples. The plates were kept at 65°C for 10-15 min to dry the DNA pellet. 200 μl TE (10 mM Tris, pH 7.5, 1 mM EDTA pH 8.0) supplemented with 3.2 μg/ml RNase, was added to each of the wells and the plates were kept at 4°C overnight for dissolution. Thereafter, the plates were centrifuged at 4700 rpm at 4°C for 30 min to pellet any undissolved material. An aliquot from the supernatant (180 μl) was transferred to fresh 1 ml plate and then sealed with a fresh sealing mat.

#### Step E: Quantification of DNA

The DNA samples were incubated at 65°C for 15 min for equal dissolution of the samples and to avoid any stratification of DNA before quantification. Estimation of the yield of isolated DNA samples was done either spectrophotometrically by Nanodrop-1000 or after electrophoresis on 1% (w/v) agarose gel by comparative fluorescence quantification of ethidium bromide stained bands using known standards. Finally, all the samples were equalized to 5 ng/μl and further diluted to 1 ng/μl for screening mutations by TILLING.

#### Mutation screening by TILLING

The standard protocol of Colbert *et al*. [6] and Till *et al *[19] was followed to detect mutations with a primer pair designed to amplify a region harboring a known mutation.

#### Primer design and amplification

Forward (5' GCTTGTTCTGGACCTTTTATGTTTGATTGG 3') and reverse primers (5' ATGAAGCACCACCAGAAATAGGCAACT 3') were designed to amplify a 901 bp region of exon 11 of UV damaged DNA binding protein 1 (*high pigment 1*) from *hp1 *mutant of tomato. The mutant, *hp1 *has an A to T transition in the gene at 11090 bp (AY531661) [29]. Upon digestion with CEL I enzyme, it generated two fragments of 718 bp and 183 bp. Amplification reaction was set up in a volume of 10 μl with 5 ng of 8×-pooled DNA having *hp1 *mutant DNA as one of the samples. The reaction consisted of 5 μl of template, 1× PCR buffer (10 mM Tris, 5 mM KCl, 1.5 mM MgCl_2_, 0.1% (w/v) gelatin, 0.005% (v/v) Tween-20, 0.005% (v/v) Np-40, pH 8.8), 0.2 mM dNTPs, 0.18 μl *Taq *polymerase (in-house isolated) and 3 pmoles of primers combined in a ratio of 3:2:4:1 (forward labeled: forward unlabeled: reverse labeled: reverse unlabeled). The cycling conditions for amplification were 94°C-5 min, 35 cycles of 94°C-20 sec, 72°C-2 min followed by heteroduplex formation: 99°C-10 min, 80°C-20 sec, 70 cycles of 80°C-7 sec with a decrement of 0.3°C per cycle and held at 4°C.

#### Mismatch cleavage and mutant detection

CEL I was isolated from celery as previously described [19,32]. The mismatch cleavage reaction was performed in a total volume of 45 μl containing 10 μl PCR product, 1× CEL I digestion buffer (10 mM HEPES buffer pH 7.0, 10 mM KCl, 10 mM MgCl_2_, 0.002% (v/v) Triton X-100 and 10 μg/ml BSA) and CEL I enzyme at 1: 300 dilution (1 μl/300 μl CEL I digestion buffer). The reaction was incubated at 45°C for 15 min and then stopped by adding 10 μl stop solution (2.5 M NaCl, 75 mM EDTA, pH 8.0 and 0.5 mg/ml blue dextran). Precipitation was done by addition of 125 μl of cold absolute ethanol and a brief incubation in -80°C followed by centrifugation at 4500 rpm in a SH-3000 rotor for 30 min. The pellet was given a 70% (v/v) ethanol wash, dried in a dry bath at 80°C and then suspended in 8 μl formamide loading buffer (37% (v/v) deionized formamide, 1 mM EDTA and 0.02% (w/v) bromophenol blue). The products were denatured by heating to 94°C for 2 min and then placed on ice. About 0.5 μl of the sample was loaded on a denaturing 6.5% (w/v) polyacrylamide gel and was electrophoresed in TBE buffer (89 mM Tris, 89 mM boric acid, 2 mM EDTA, pH 8.3) at 1500 V, 40 mA and 40 V setting on 4300 Li-COR Analyzer. The two TIFF images of 700 and 800 channels were analyzed in Adobe Photoshop software (Adobe Systems Inc.) and the gel was visually assessed for mutations.

## Competing interests

The authors declare that they have no competing interests.

## Authors' contributions

YS and RS did overall conceptualization of cotyledon pooling from different plants, evaluation and modification of DNA isolation protocols. The data for different figures and table were contributed as follows: Figure 1 and Table 1 (VSC), Figure 2 (YS, SS), Figure 3 and 7 (RB), Figure 4 (HM, ST, VM- equal contribution), Figure 5 and 6 (SG). Figure 4 was drawn by SG. Rajeswari Srinivasan (RaS) contributed to initial evaluation of extraction conditions. SG, YS and RS were involved in writing of the manuscript. All authors read and approved the manuscript.
